# Utilizing Negative Pressure Wound Therapy with Instillation and Dwell Time for Extensive Necrotizing Fasciitis of the Lower Extremity: A Case Report

**DOI:** 10.7759/cureus.3483

**Published:** 2018-10-23

**Authors:** John P Livingstone, Ian G Hasegawa, Patrick Murray

**Affiliations:** 1 Orthopedic Surgery, Queen's Medical Center, Honolulu, USA; 2 Orthopedic Surgery, University of Hawaii, John A. Burns School of Medicine, Honolulu, USA

**Keywords:** necrotizing, fasciitis, negative, pressure, wound, therapy, instillation, dwell, infection, extremity

## Abstract

Necrotizing fasciitis is a rapidly spreading infection of the soft tissue, which carries significant morbidity and mortality. This condition is treated with broad-spectrum antibiotics, irrigation and surgical debridement of the affected area, and hemodynamic support. Negative pressure wound therapy (NPWT) has been utilized after surgical debridement to promote wound healing, especially when significant debridement has occurred. Newer forms of NPWT such as negative pressure wound therapy with instillation and dwell time (NPWTi-d) have shown even greater promise by reducing the time to clear infections and promoting greater debridement with fewer procedures. This case report demonstrates the successful use of NPWTi-d on a 56-year-old man with a severe case of necrotizing fasciitis of the right lower extremity after extensive debridement. Despite the significant loss of soft tissue and the circumferential devitalization of the lower leg, this patient was able to accept a skin graft in approximately four weeks after admission to the hospital. Three months after initial presentation, his wounds were completely epithelialized and healing well. The advantages of using NPWTi-d include decreased dressing changes, increased wound granulation, and faster infection clearance. The disadvantages of such systems include increased cost, additional technical requirements, and required inpatient monitoring of the system. Despite these disadvantages, the authors believe that NPWTi-d is a reasonable choice for patients similar to the one presented in this case report.

## Introduction

Necrotizing fasciitis, commonly publicized as flesh-eating bacteria, is a soft tissue infection with rapidly spreading necrosis and inflammation of the skin, subcutaneous fat, and fascia [1]. The diagnosis of necrotizing fasciitis carries significant morbidity and mortality with some studies reporting mortality rates as high as 20% [2-4]. The gold standard of treatment for necrotizing fasciitis includes early surgical irrigation and debridement, broad-spectrum antibiotics, nutritional support, and hemodynamic support [5-6]. With the advent of negative pressure wound therapy (NPWT) over 20 years ago [7], physicians have more recently begun to treat necrotizing fasciitis with NPWT as well.

NPWT is a method of wound management that involves placing an occlusive dressing over specialized wound packing and applying a vacuum to the dressing. This has been shown to promote wound healing, manage wound drainage, and prevent infection. NPWT has also been shown to increase cell proliferation, increase local perfusion, increase granulation, clear wound infections earlier, decrease hospital length of stay, and expedite final wound closure [8]. NPWT is commonly used for chronic wounds such as pressure ulcers or diabetic wounds but can also be used for traumatic or surgical wounds. Newer negative pressure wound therapy systems instill a set volume of solution into the wound, allow the solution to dwell, and then use negative pressure to remove the fluid and any debris along with it [9]. These systems are classified as negative pressure wound therapy systems with instillation and dwell time (NPWTi-d). NPWTi-d has further improved NPWT technology by decreasing bioburden, hospital length of stay, time to final wound closure, and need for surgical debridements in comparison to traditional NPWT [8-9]. NPWTi-d can be used in most instances where NPWT would be used. It is especially useful for infected wounds requiring frequent debridement.

Several studies have shown the beneficial effects of NPWT in necrotizing fasciitis [5,10-14]. However, there is limited research on using NPWTi-d for necrotizing fasciitis, with only four cases currently published [15-18]. This case report demonstrates the successful use of NPWTi-d in a case of necrotizing fasciitis in the right lower extremity, after extensive debridement.

## Case presentation

A 56-year-old homeless male with a history of hypertension and a seizure disorder presented to the emergency department with a one-day history of pain, redness, and swelling of the right lower extremity. In the emergency department, the patient was febrile with a temperature of 102˚F and otherwise had normal vital signs. His physical exam revealed diffuse erythema and swelling to the right lower extremity extending up to the medial aspect of the right thigh. The patient was admitted to the hospital and was started on intravenous cefazolin, intravenous vancomycin, and oral clindamycin.

**Figure 1:**
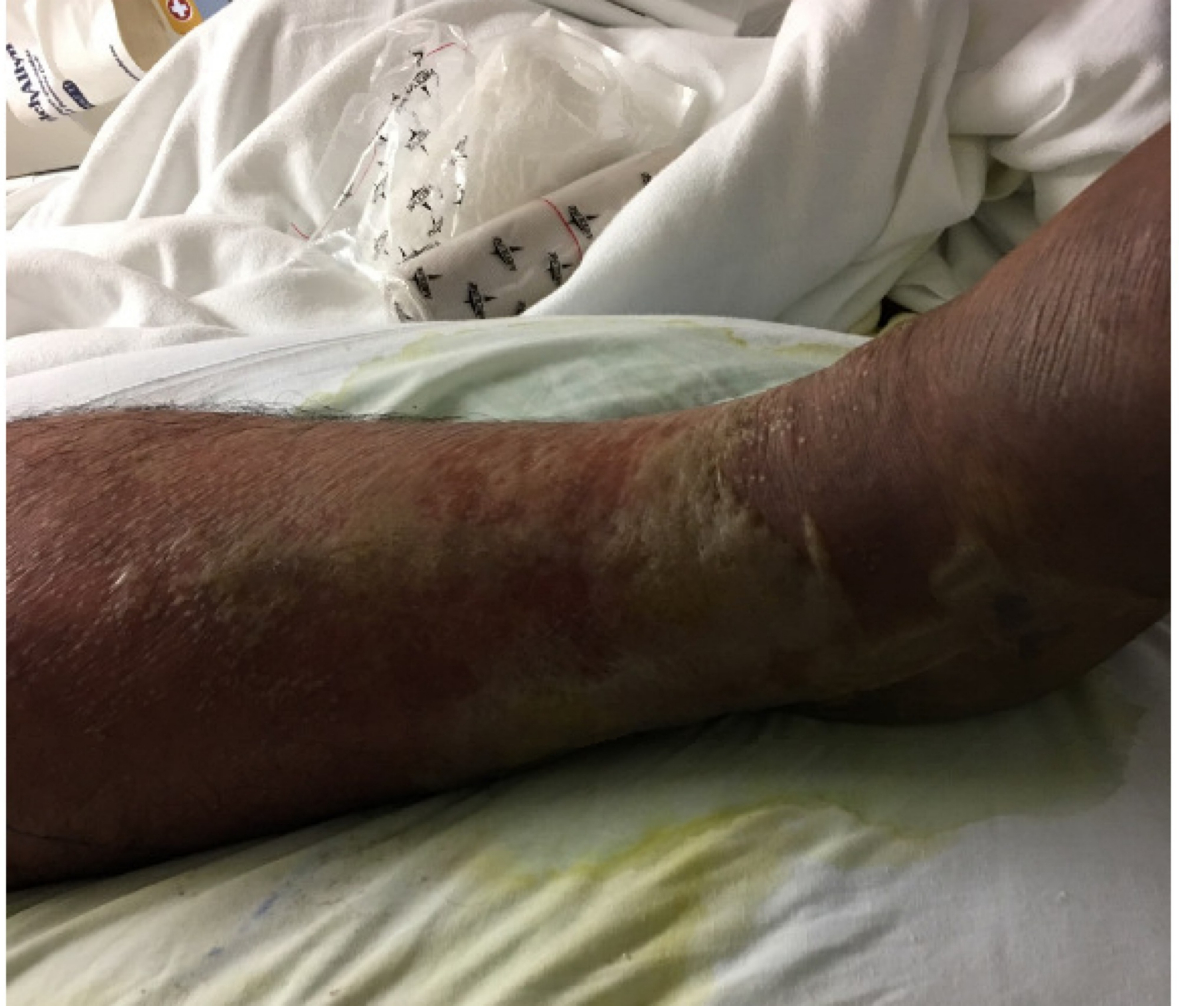
Right lower extremity on hospital Day 3

**Figure 2:**
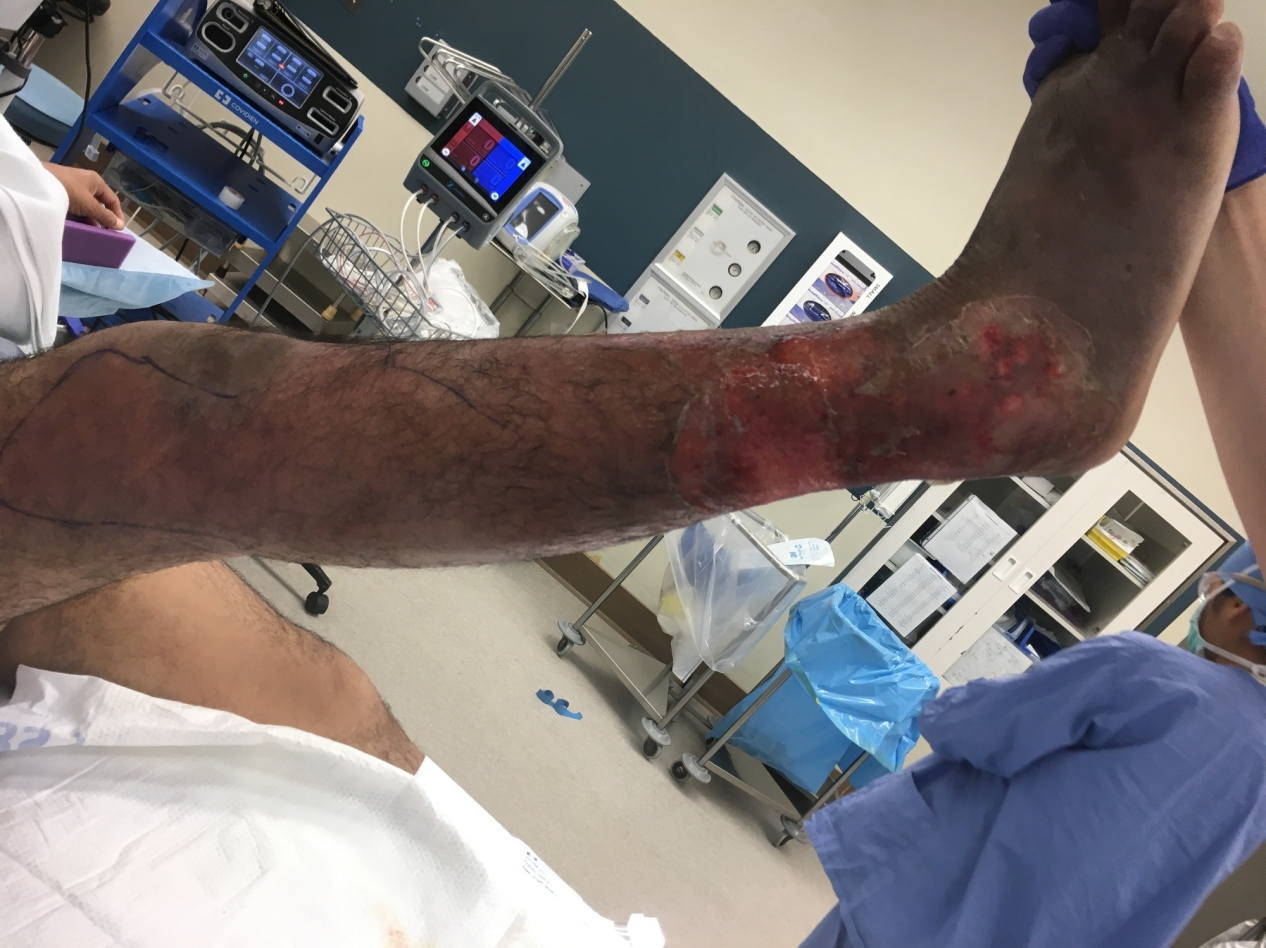
Right lower extremity; hospital Day 4 prior to debridement

**Figure 3:**
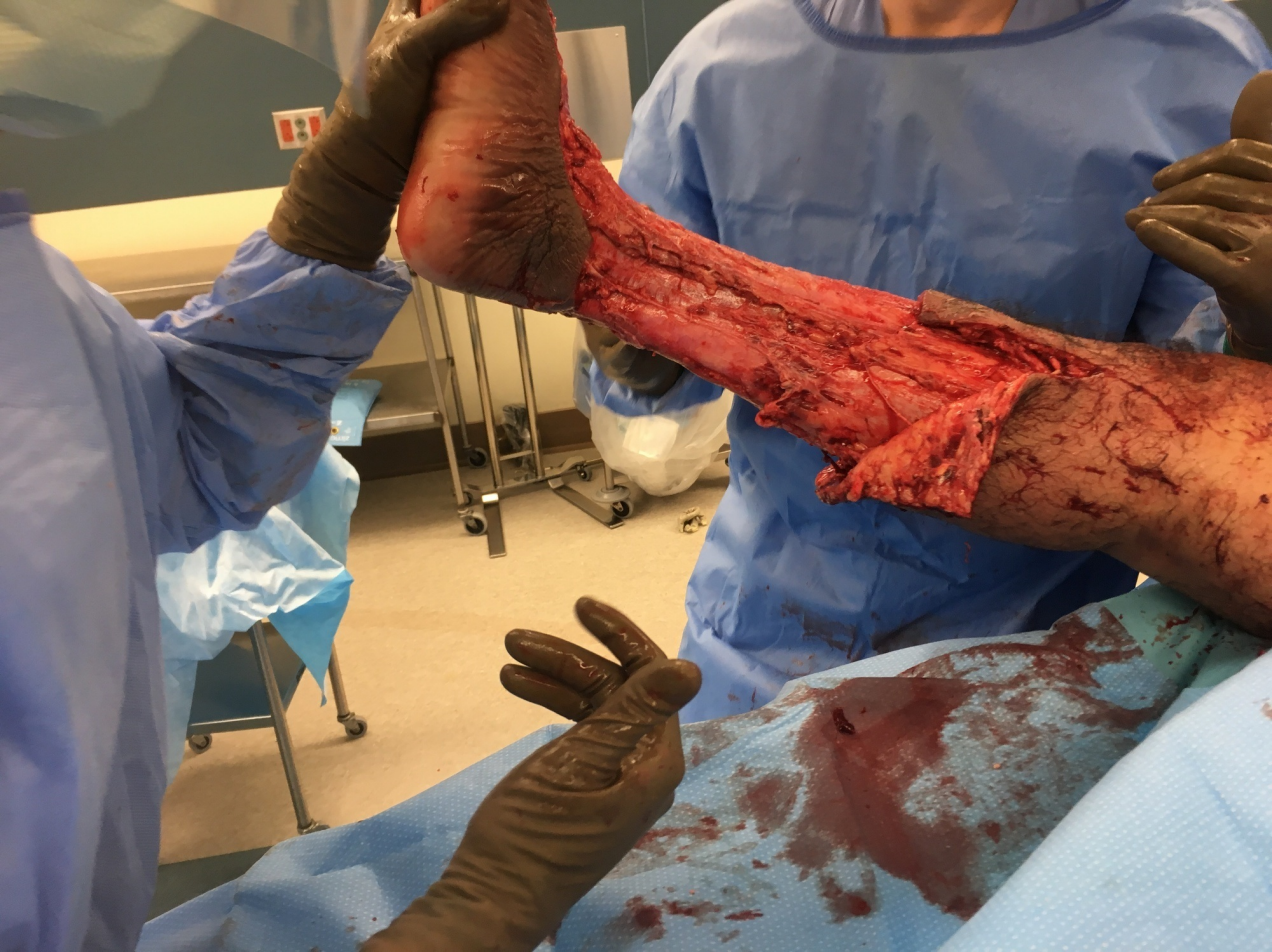
Medial view of right lower extremity after initial debridement

**Figure 4:**
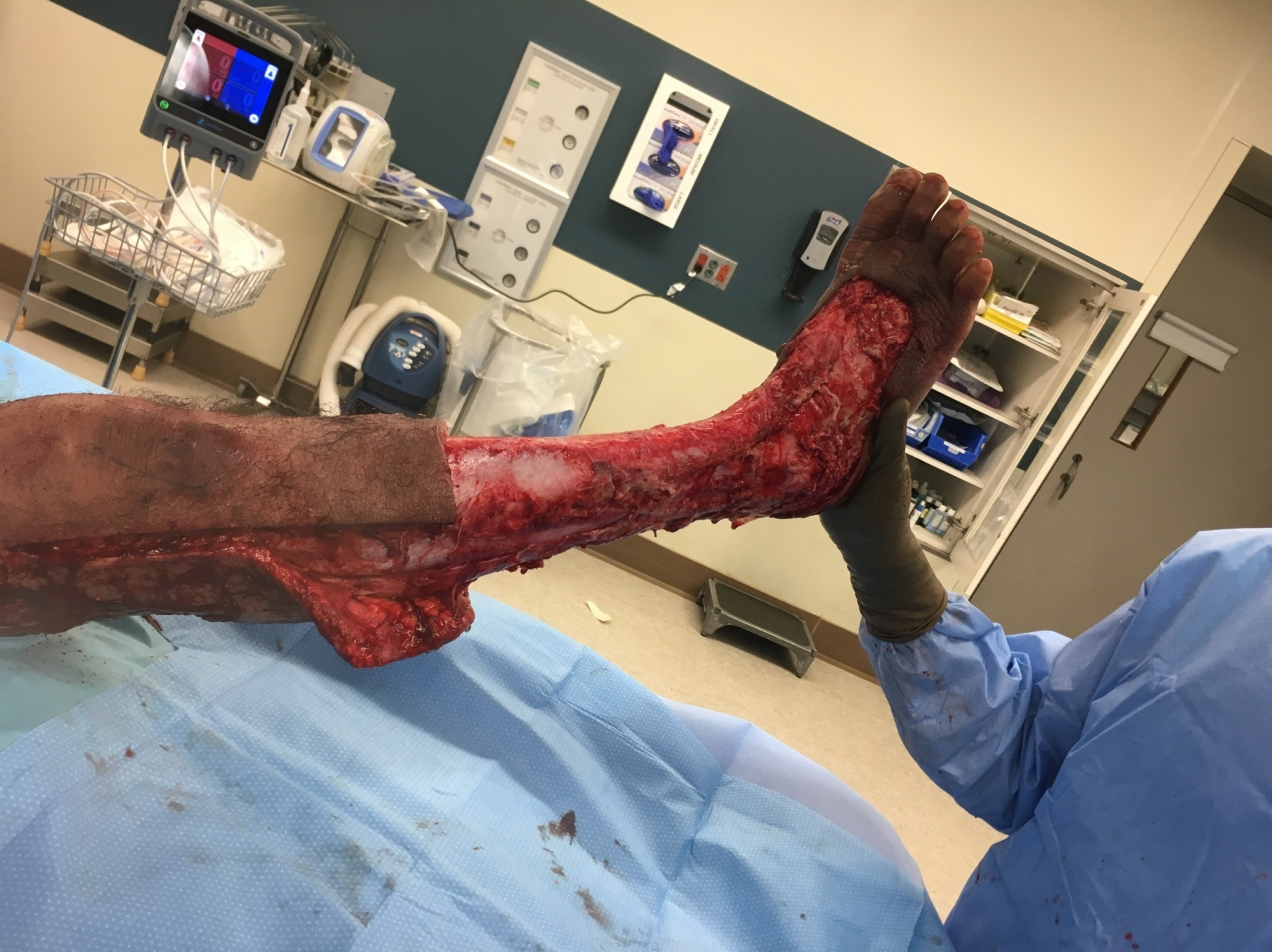
Lateral view of right lower extremity after initial debridement

**Figure 5:**
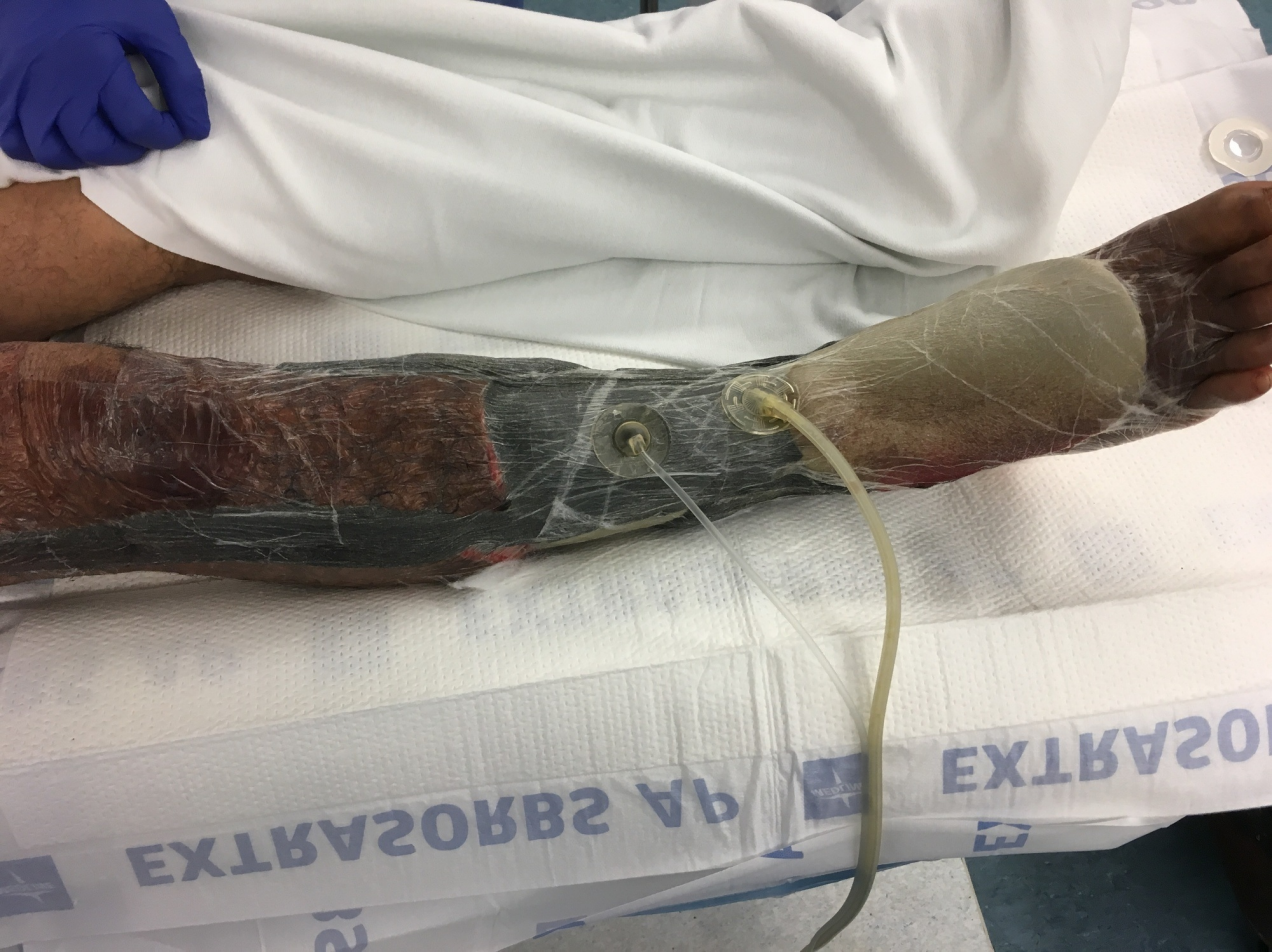
Right lower extremity after placement of NPWTi-d NPWTi-d: negative pressure wound therapy with instillation and dwell time

**Figure 6:**
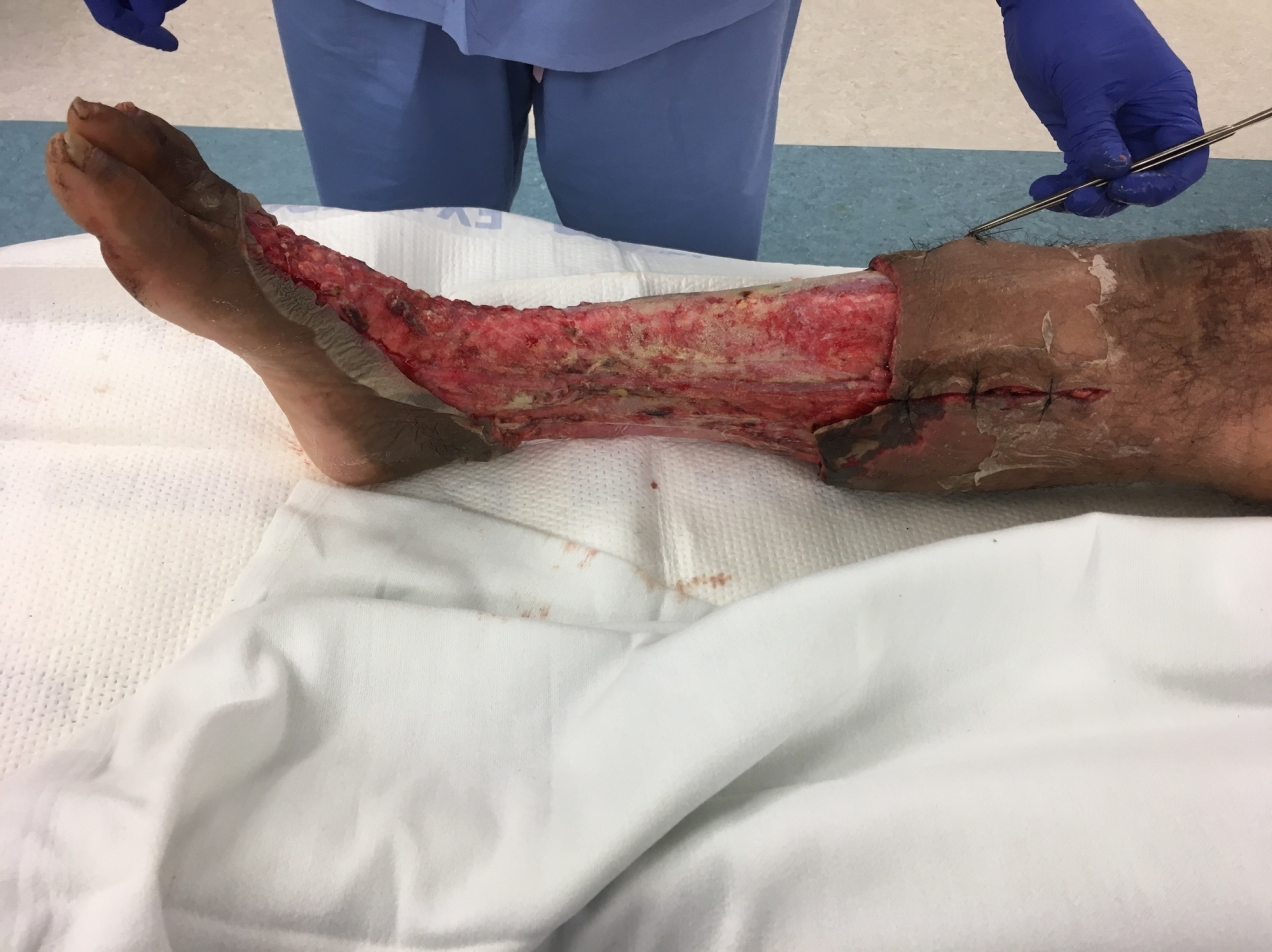
Medial view of right lower extremity; hospital Day 8 during first NPWTi-d change NPWTi-d: negative pressure wound therapy with instillation and dwell time

**Figure 7:**
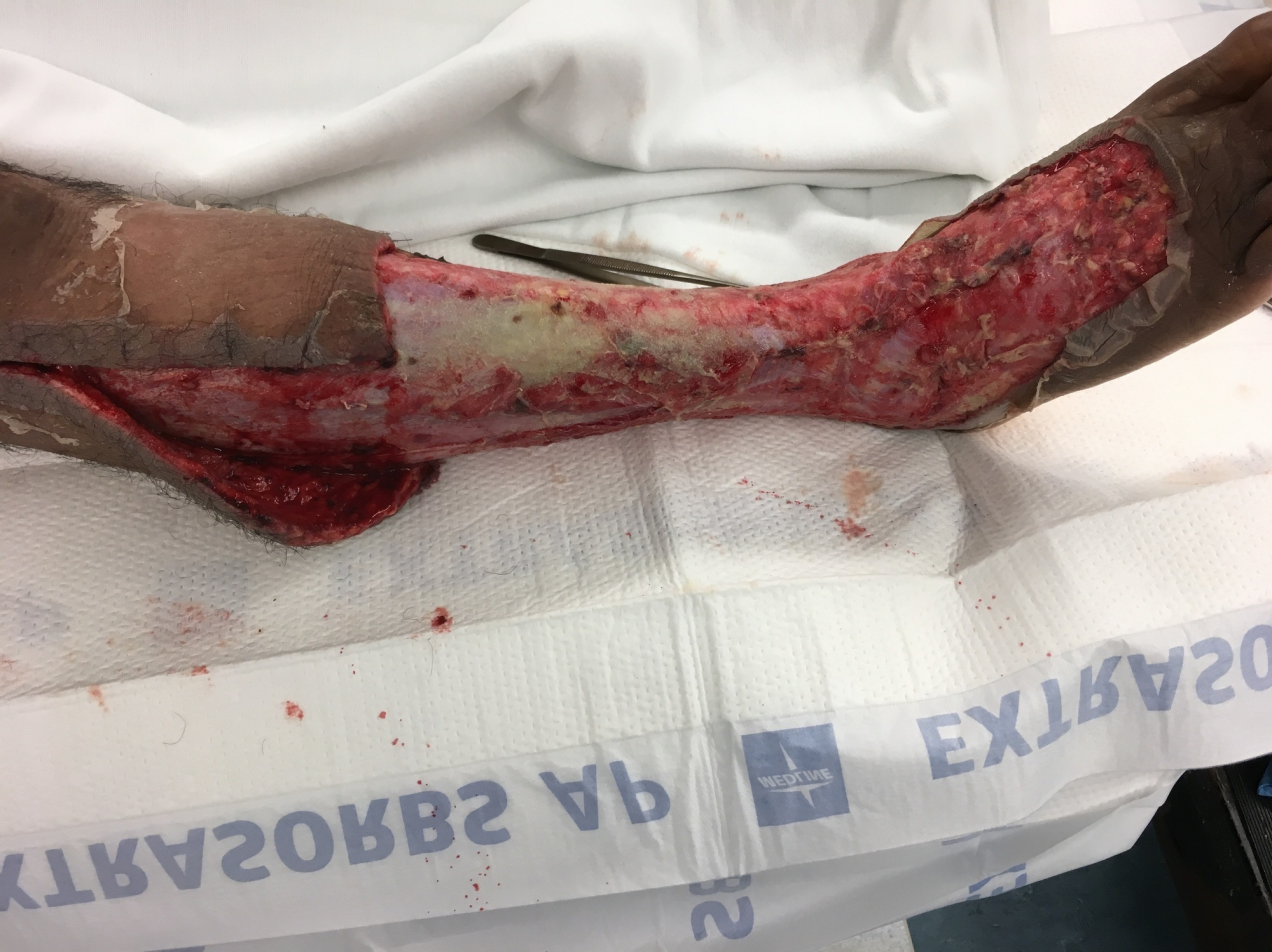
Lateral view of right lower extremity; hospital Day 8 during first NPWTi-d change NPWTi-d: negative pressure wound therapy with instillation and dwell time

**Figure 8:**
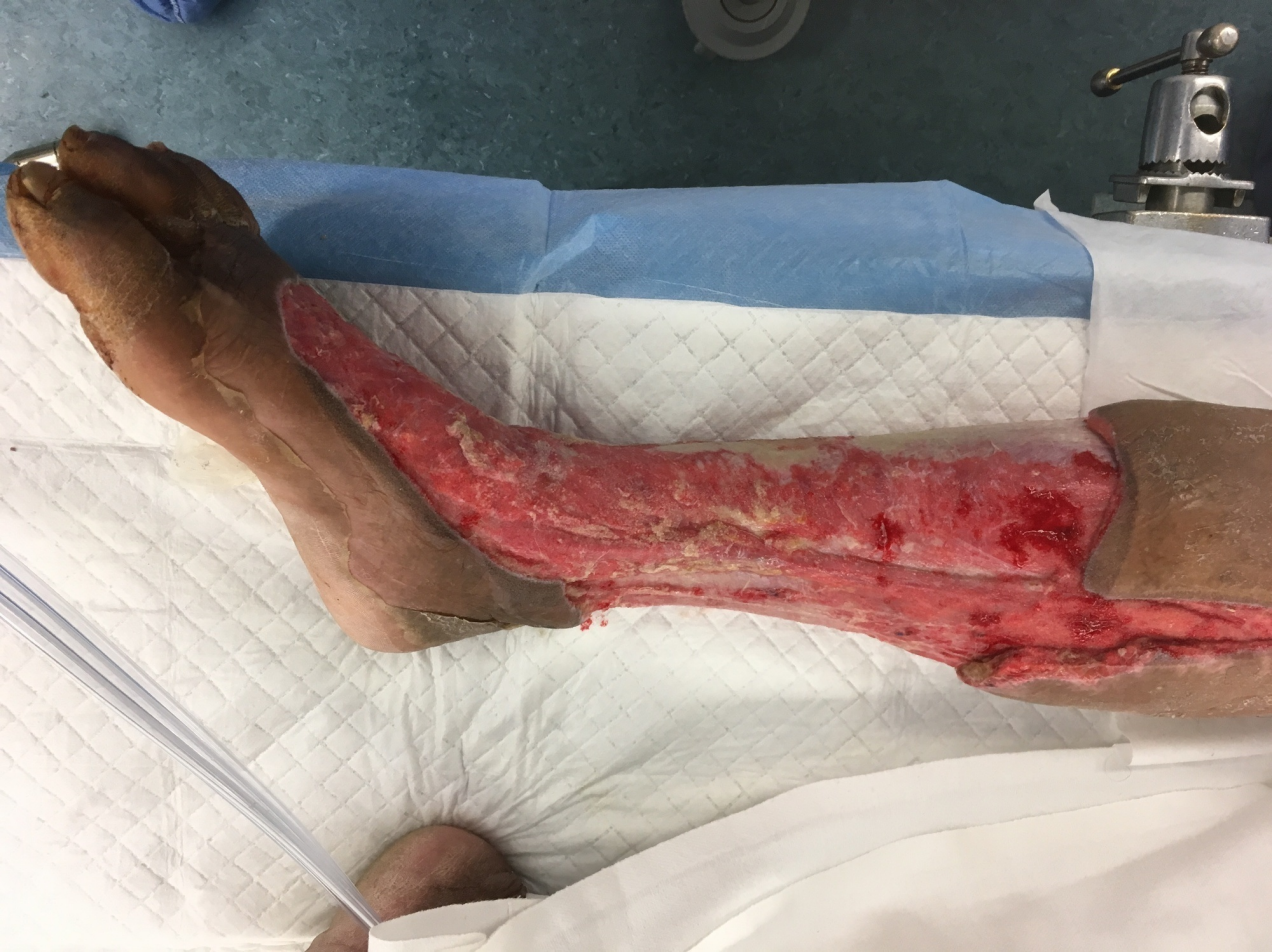
Medial view of right lower extremity; hospital Day 16 during second NPWTi-d change NPWTi-d: negative pressure wound therapy with instillation and dwell time

**Figure 9:**
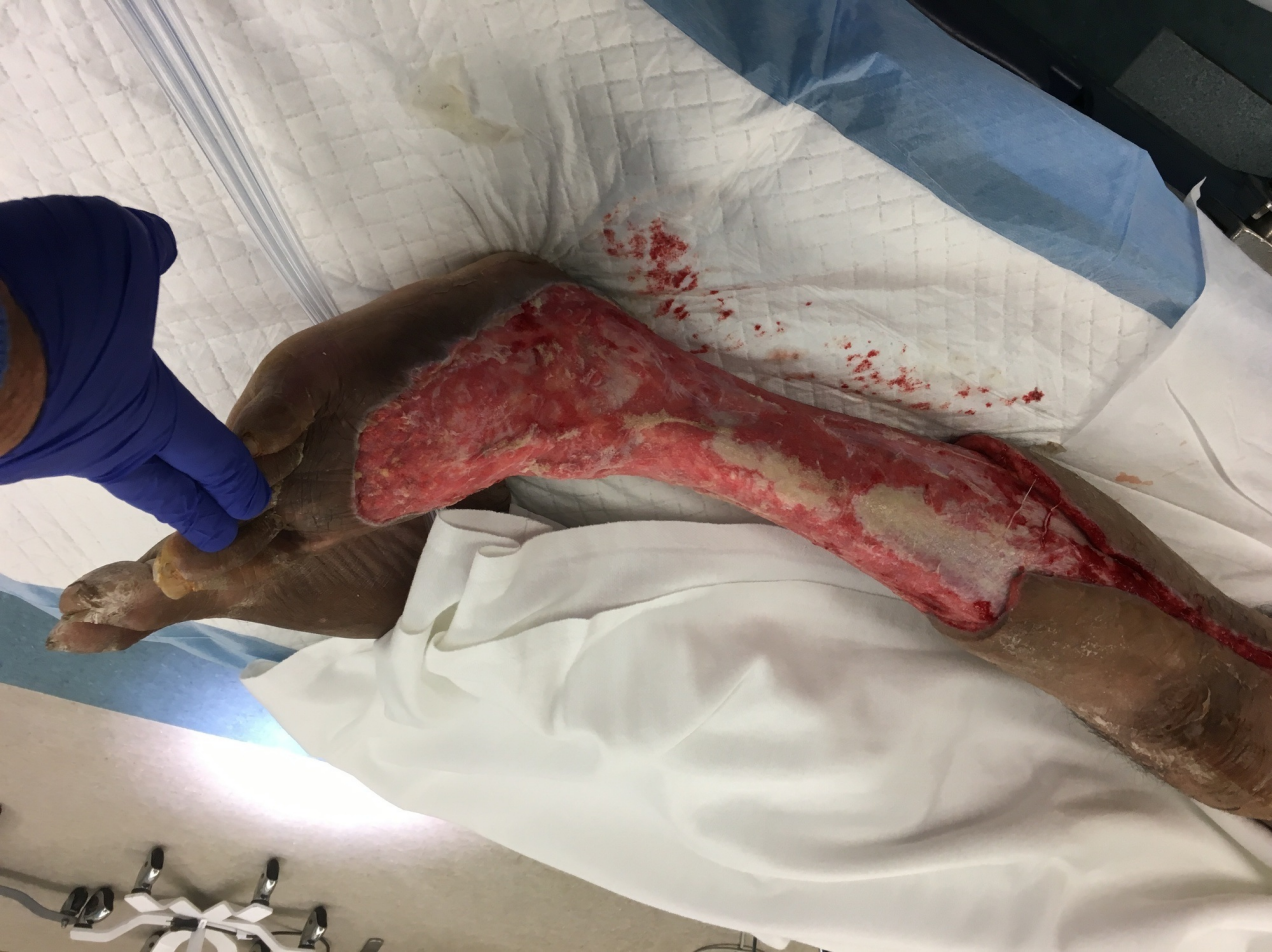
Lateral view of right lower extremity; hospital Day 16 during second NPWTi-d change NPWTi-d: negative pressure wound therapy with instillation and dwell time

**Figure 10:**
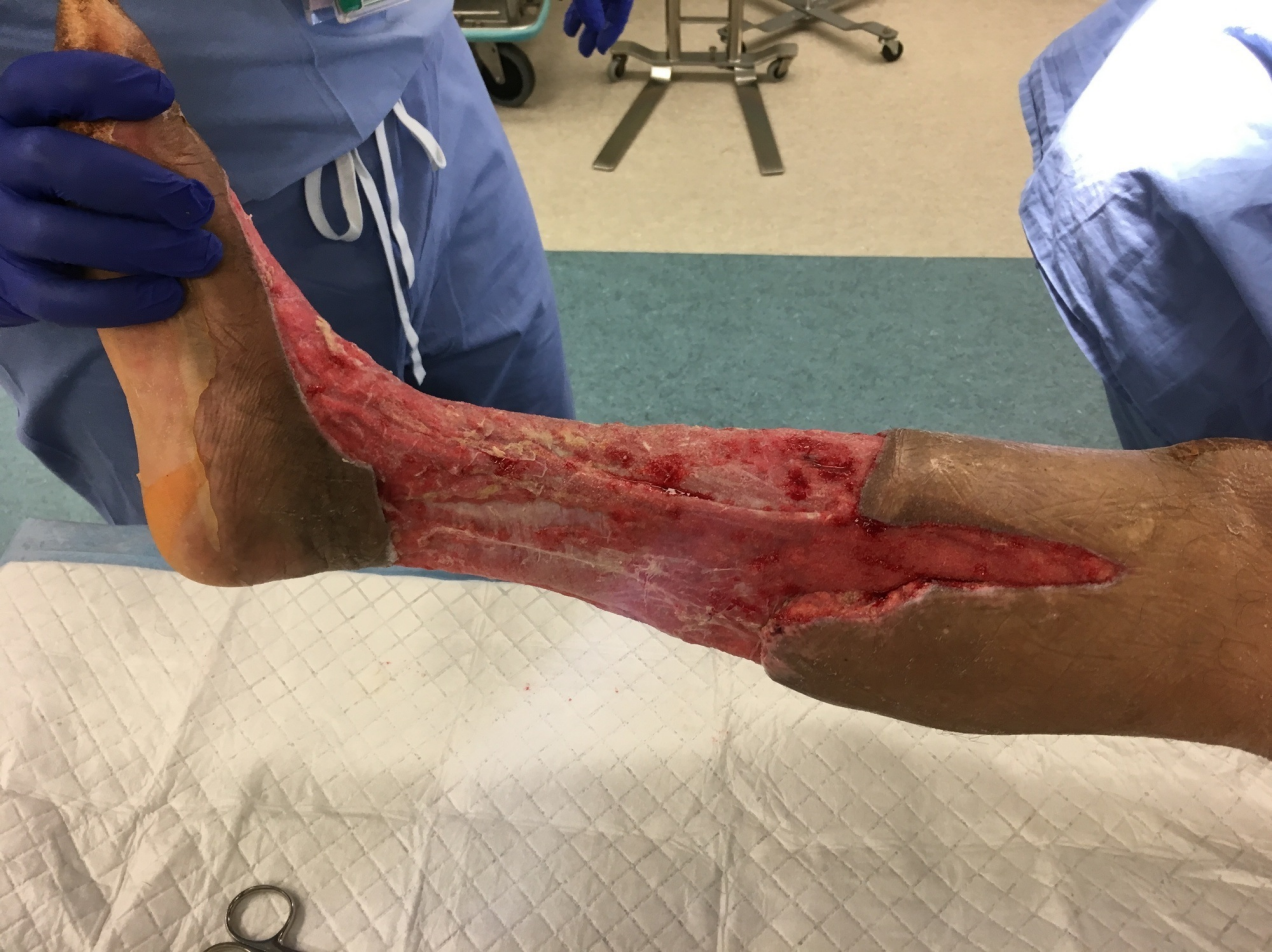
Medial view of right lower extremity; hospital Day 22 during third NPWTi-d change NPWTi-d: negative pressure wound therapy with instillation and dwell time

**Figure 11:**
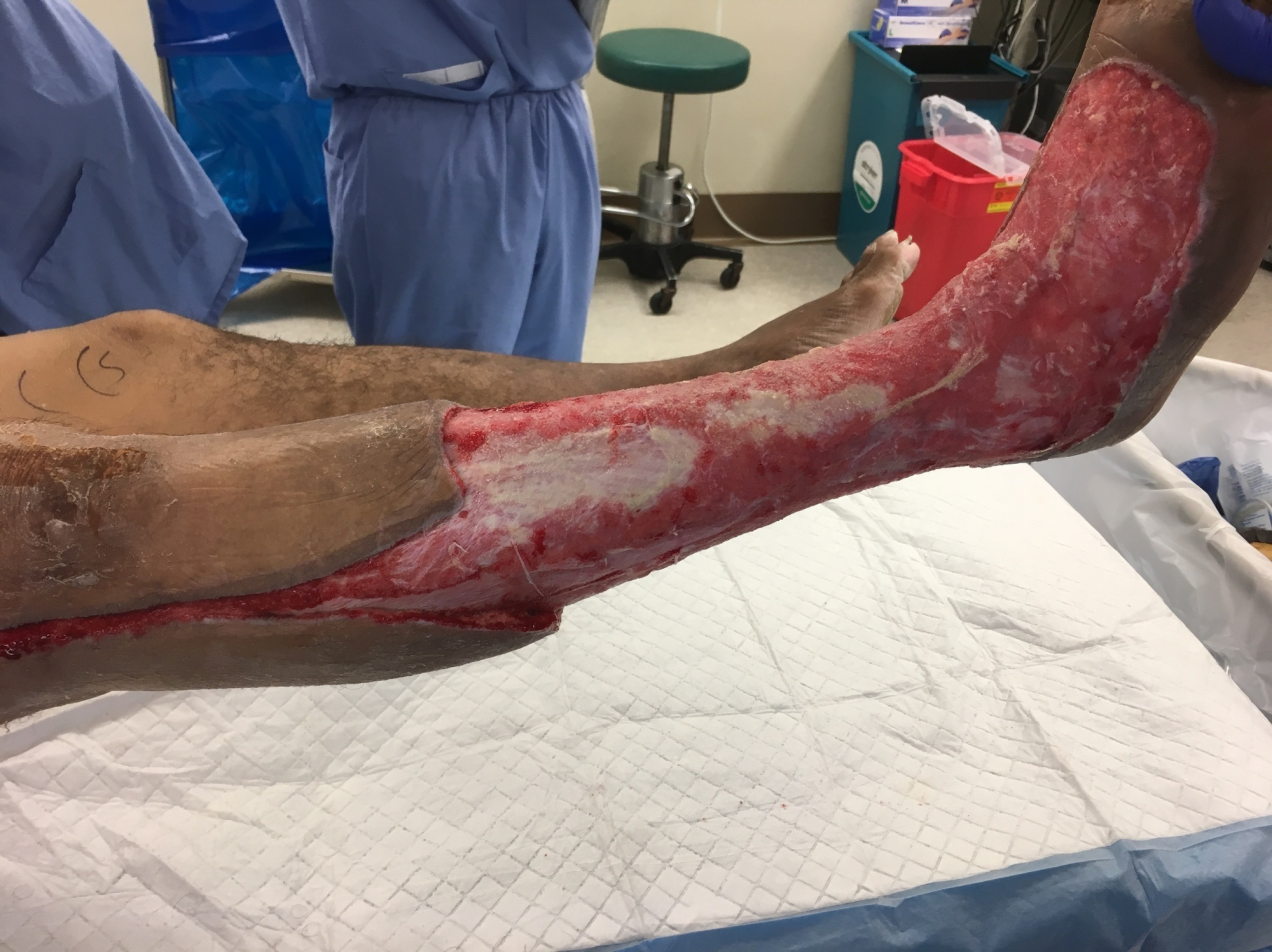
Lateral view of right lower extremity; hospital Day 22 during third NPWTi-d change NPWTi-d: negative pressure wound therapy with instillation and dwell time

**Figure 12:**
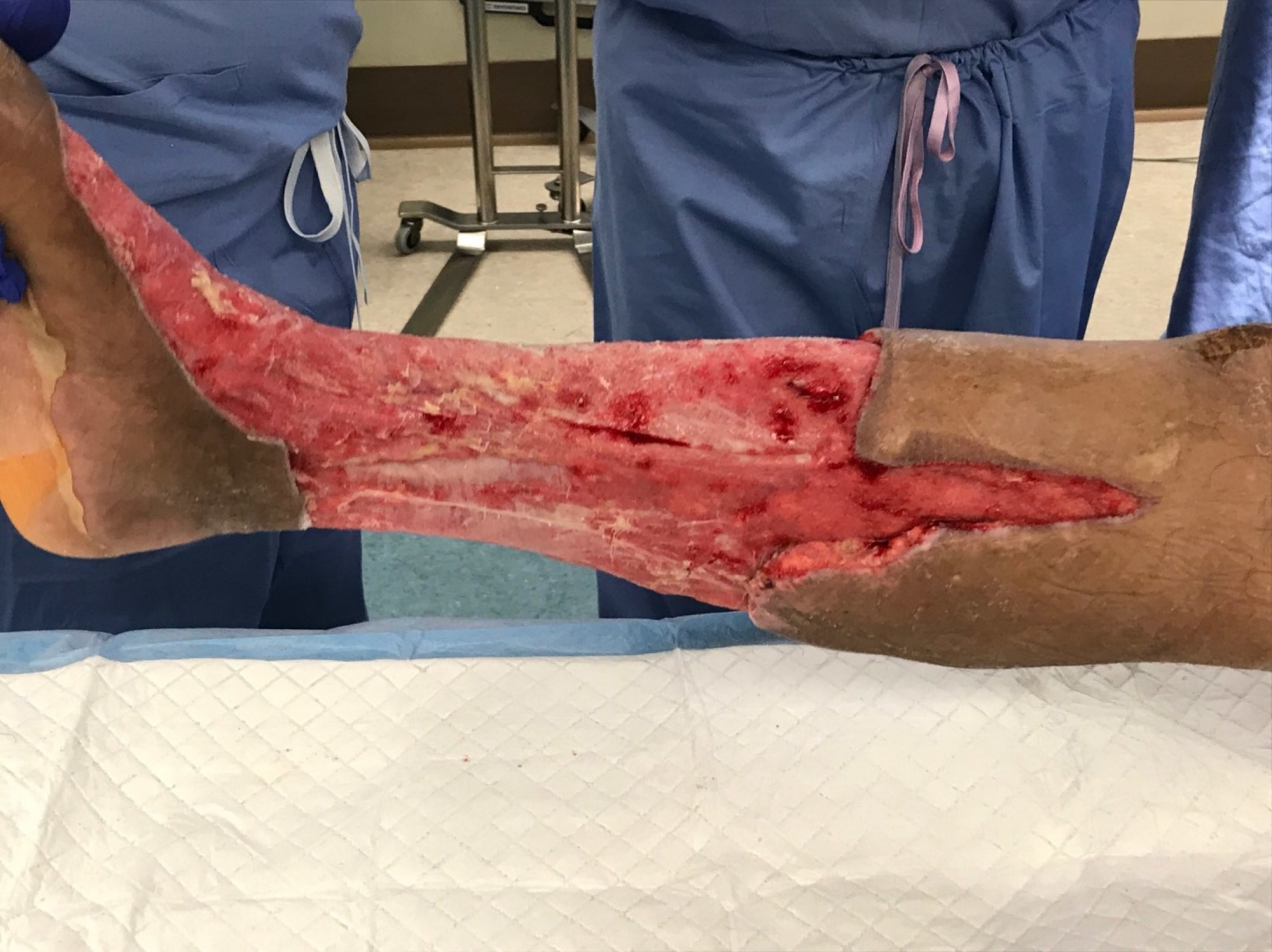
Right lower extremity; hospital Day 29 prior to skin grafting

Despite antibiotic therapy and bedside debridement, the patient’s erythema continued to spread (see Figure 1). On hospital Day 4, it was determined that the patient required irrigation and debridement of his right lower extremity wound. Extensive soft tissue debridement was required as a large portion of the right lower extremity was found to be necrotic (see Figure 2). Devitalized tissue was removed from the dorsum of the foot, circumferentially around the lower leg and laterally to the level of the knee (see Figures 3-4). An NPWTi-d device was applied over the wound in the operating room (see Figure 5). A special three-layer reticulated open cell foam dressing was applied over the dorsum of the foot where the most necrotic tissue was found prior to debridement. This reticulated open cell foam is designed to promote additional debridement. The more proximal wounds were covered with a standard reticulated open cell foam that is designed primarily to promote granulation tissue formation. The wound was irrigated with Prontosan for the first three days after placement of the NPWTi-d device to assist with wound debridement via its surfactant properties. Initial NPWTi-d settings were -125 mmHg with a 2.5 hr negative pressure cycle, 50 mL of instillation, and a 10-minute dwell time. After three days of Prontosan instillation, normal saline was used as an instillation solution to simulate a more physiologic environment that we believe is more conducive to wound healing. The NPWTi-d device was replaced in the operating room every seven days until the wound was sufficiently granulated (see Figures 6-12). Although the manufacturer recommendations are to replace the NPWTi-d device every two to three days, we have seen similar results with replacing these devices once weekly, and our patients prefer the less frequent device changes. After each NPWTi-d replacement, Prontosan was once again used for three days and was then replaced with normal saline.

**Figure 13:**
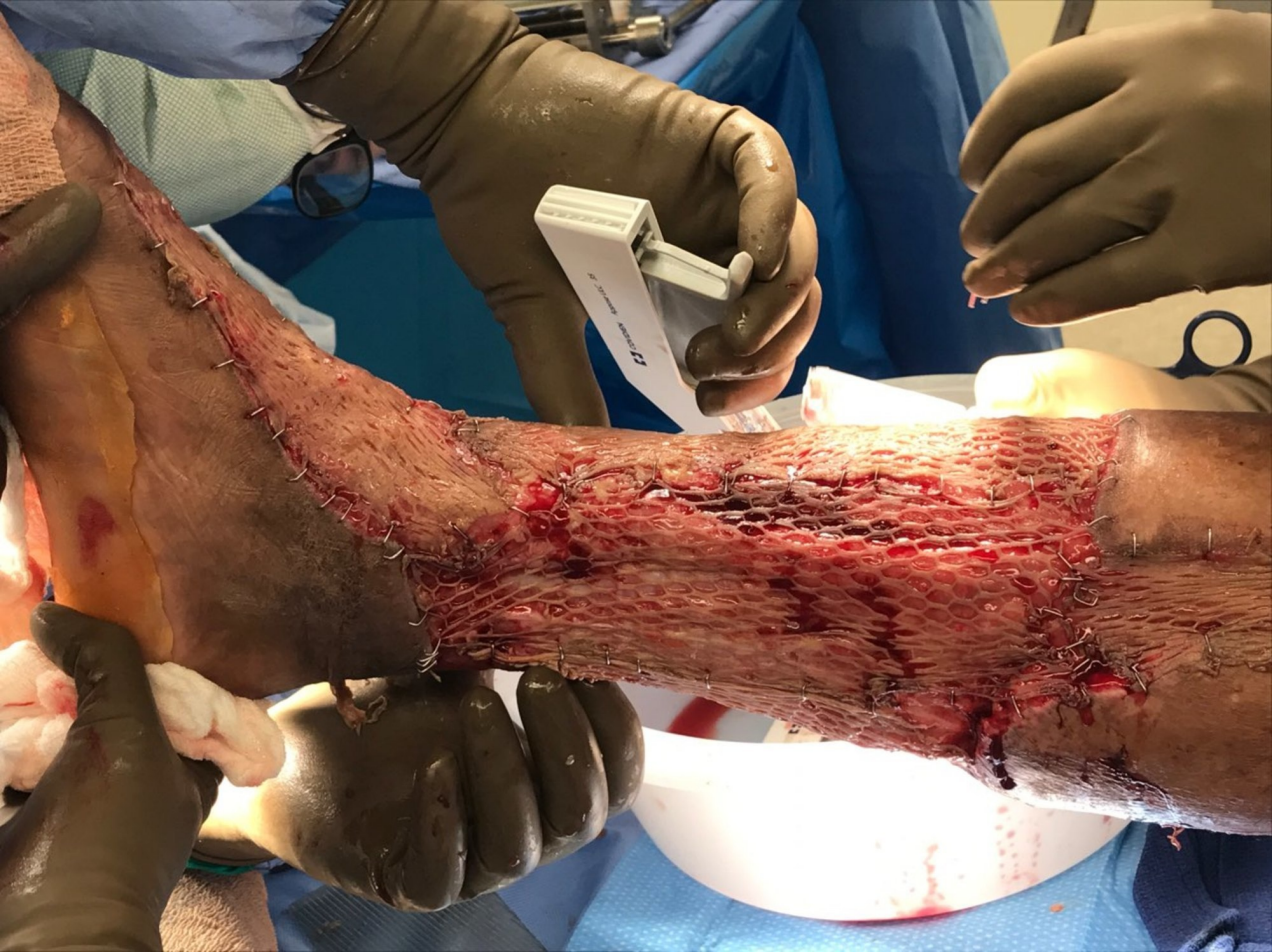
Right lower extremity; hospital Day 29 during skin grafting

**Figure 14:**
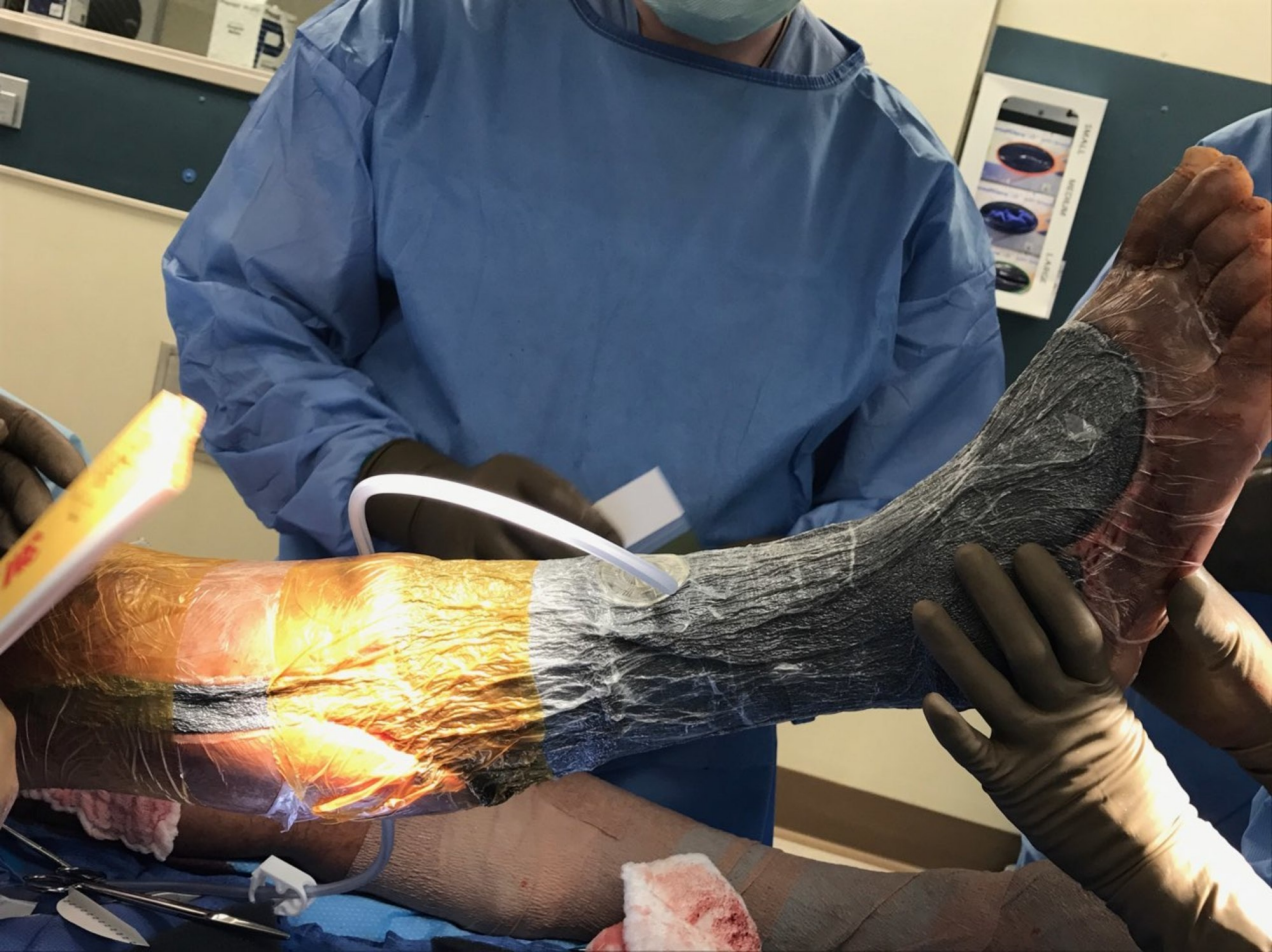
Right lower extremity; hospital Day 29 after skin grafting and placement of a negative pressure wound therapy device over the skin graft

**Figure 15:**
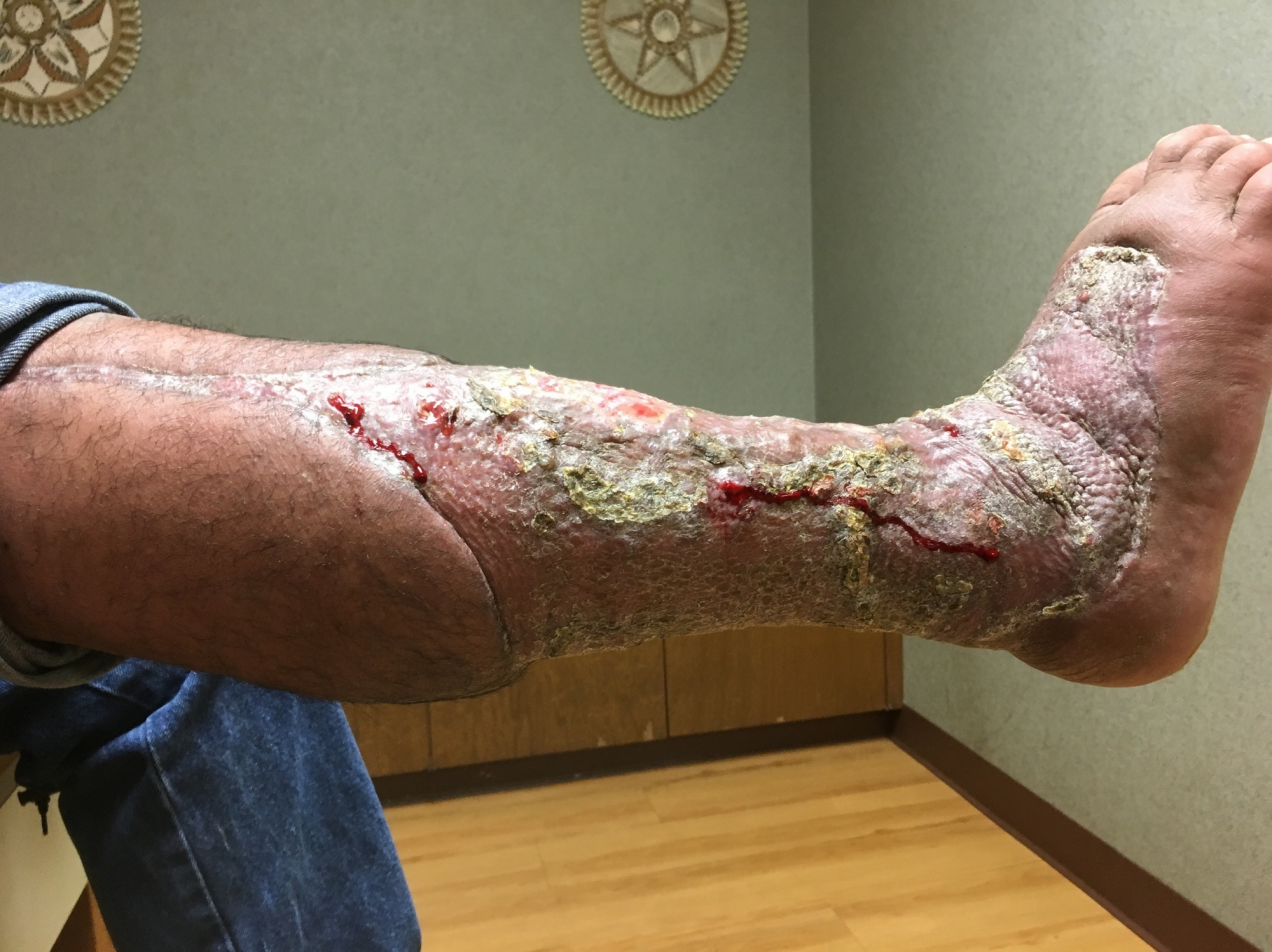
Lateral view of right lower extremity; three months after initial presentation

**Figure 16:**
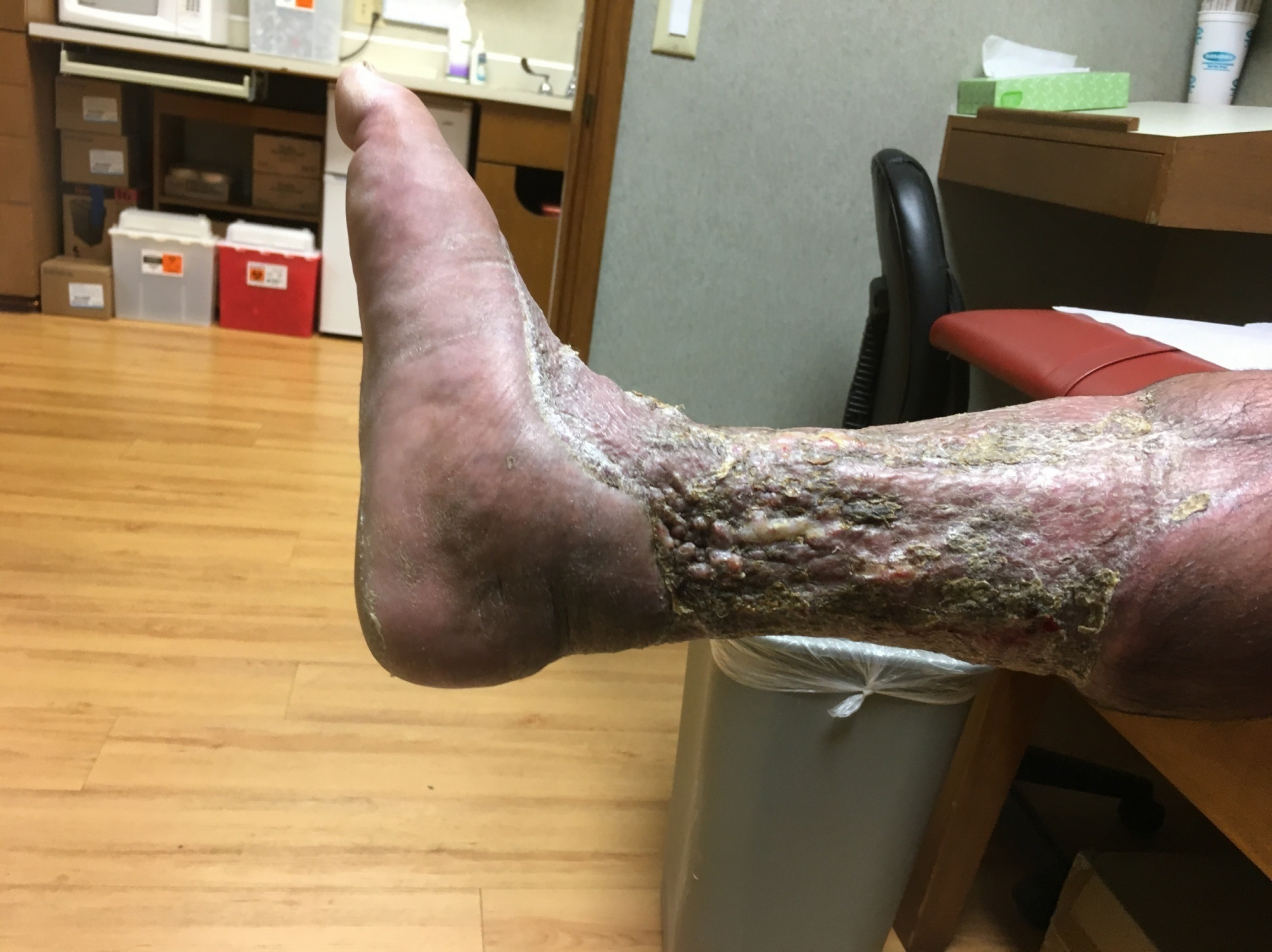
Medial view of right lower extremity; three months after initial presentation

On hospital Day 29, a skin graft from the right and left thighs were used to cover the wound and a negative pressure wound therapy device was placed over the skin graft (see Figures 13-14). On hospital Day 30, his intravenous antibiotics were discontinued. The patient was discharged on hospital Day 34 to a transitional home for homeless patients requiring additional wound care and social services. Throughout the patient's hospital admission, he was on a regular diet and maintained a body mass index (BMI) of approximately 30 throughout his stay. He did not require any oral antibiotics on discharge. The patient was followed closely with outpatient appointments thereafter. Approximately three months after the initial presentation to the emergency department, the patient was seen in the clinic for a wound check. His right lower extremity wounds were epithelialized and healing well (see Figures 15-16).

## Discussion

The use of the NPWTi-d system for this patient with necrotizing fasciitis of the right lower extremity proved to be highly successful. Despite the significant loss of soft tissue and circumferential devitalization of the lower leg, this patient was able to accept a skin graft in approximately four weeks after admission to the hospital. There are several advantages to using NPWTi-d systems in patients similar to the case reported here. First, patients require less dressing changes in comparison to traditional wet to dry dressings. This patient only required once-weekly dressing changes during his hospital course, which reduces overall patient discomfort. More importantly, NPWTi-d has been shown to increase the granulation of the treated area, decrease time to infection clearance, and decrease the length of time required for surgical closure of the affected area [8-9,19]. Disadvantages to using NPWTi-d include the higher cost of the device in comparison to traditional dressings, the additional technical expertise required by physicians and nursing staff for NPWTi-d application and management, and the inability for at-home care since NPWTi-d requires inpatient monitoring. The inpatient requirements for NPTWi-d were not problematic for this patient since he was homeless and required inpatient management regardless. However, in patients who are capable of managing their wounds at home and following up on an outpatient basis, they may benefit from outpatient battery-powered NPWT devices rather than NPWTi-d.

There have only been four published cases of necrotizing fasciitis treated with NPWTi-d [15-18]. Of these cases, only one was used on a lower extremity [15]. Unfortunately, the patient expired due to pneumonia prior to the final closure of the treated wound, making this the only published case of a lower extremity necrotizing fasciitis successfully treated with NPWTi-d. Although this is a study of a single patient’s outcomes with NPWTi-d, the authors of this paper believe that NPWTi-d is an effective treatment strategy for necrotizing fasciitis. Future studies directly comparing NPWTi-d with either NPWT or more traditional dressings in patients with necrotizing fasciitis are required to further improve our understanding of the risks and benefits of this treatment modality.

## Conclusions

NPWTi-d systems can be effectively utilized in cases of necrotizing fasciitis after extensive surgical debridement. Despite the additional costs and requirements of NPWTi-d, the authors believe that the advantages of the system significantly outweigh the disadvantages, making it a reasonable choice for patients similar to the one presented in this case report.
